# Viral hepatitis and HIV-associated tuberculosis: Risk factors and TB treatment outcomes in Thailand

**DOI:** 10.1186/1471-2458-8-245

**Published:** 2008-07-18

**Authors:** Chawin Sirinak, Wanitchaya Kittikraisak, Duangporn Pinjeesekikul, Pricha Charusuntonsri, Phinai Luanloed, La-ong Srisuwanvilai, Sriprapa Nateniyom, Somsak Akksilp, Sirirat Likanonsakul, Wanchai Sattayawuthipong, Channawong Burapat, Jay K Varma

**Affiliations:** 1 Department of Health, Bangkok Metropolitan Administration, Bangkok, Thailand; 2 Thailand Ministry of Public Health – U.S. Centers for Disease Control and Prevention Collaboration, Nonthaburi, Thailand; 3 Thailand Ministry of Public Health, Nonthaburi, Thailand; 4 Office of Disease Prevention and Control 7, Ubon-ratchathani, Thailand; 5 Bamrasnaradura Infectious Diseases Institute, Nonthaburi, Thailand; 6 Phuket Provincial Health Office, Phuket, Thailand; 7 U.S. Centers for Disease Control and Prevention, Atlanta, USA

## Abstract

**Background:**

The occurrence of tuberculosis (TB), human immunodeficiency virus (HIV), and viral hepatitis infections in the same patient poses unique clinical and public health challenges, because medications to treat TB and HIV are hepatotoxic. We conducted an observational study to evaluate risk factors for HBsAg and/or anti-HCV reactivity and to assess differences in adverse events and TB treatment outcomes among HIV-infected TB patients.

**Methods:**

Patients were evaluated at the beginning, during, and at the end of TB treatment. Blood samples were tested for aspartate aminotransferase (AST), alanine aminotransferase (ALT), total bilirubin (BR), complete blood count, and CD4+ T lymphocyte cell count. TB treatment outcomes were assessed at the end of TB treatment according to international guidelines.

**Results:**

Of 769 enrolled patients, 752 (98%) had serologic testing performed for viral hepatitis: 70 (9%) were reactive for HBsAg, 237 (31%) for anti-HCV, and 472 (63%) non-reactive for both markers. At the beginning of TB treatment, 18 (26%) patients with HBsAg reactivity had elevated liver function tests compared with 69 (15%) patients non-reactive to any viral marker (p = 0.02). At the end of TB treatment, 493 (64%) were successfully treated. Factors independently associated with HBsAg reactivity included being a man who had sex with men (adjusted odds ratio [AOR], 2.1; 95% confidence interval [CI], 1.1–4.3) and having low TB knowledge (AOR, 1.8; CI, 1.0–3.0). Factors most strongly associated with anti-HCV reactivity were having injection drug use history (AOR, 12.8; CI, 7.0–23.2) and living in Bangkok (AOR, 15.8; CI, 9.4–26.5). The rate of clinical hepatitis and death during TB treatment was similar in patients HBsAg reactive, anti-HCV reactive, both HBsAg and anti-HCV reactive, and non-reactive to any viral marker.

**Conclusion:**

Among HIV-infected TB patients living in Thailand, markers of viral hepatitis infection, particularly hepatitis C virus infection, were common and strongly associated with known behavioral risk factors. Viral hepatitis infection markers were not strongly associated with death or the development of clinical hepatitis during TB treatment.

## Background

Over nine million new cases of tuberculosis (TB) occur annually throughout the world. While most of these cases can safely and effectively be treated, complications can occur during TB treatment, because of anti-TB drug resistance, poor adherence, drug-drug interactions, and toxicity.[1] Liver toxicity is a particularly common side effect that is strongly associated with three of the four anti-TB drugs included in the most widely accepted regimen.[2,3] The burden of TB continues to rise in some regions of the world, because of the human immunodeficiency virus (HIV) epidemic. This TB/HIV syndemic has also increased the clinical complexity of managing patients. Medications used to prevent opportunistic infections or treat HIV are often hepatotoxic, and opportunistic infections may involve the liver. [4-6]

The epidemics of hepatitis B and/or C virus infection (HBV and HCV, respectively) involve many of the populations that are at risk of HIV infection. This is particularly true in Eastern Europe and Asia, where injection drug use is a potent driver of the HIV epidemic. [7-9] The occurrence of TB, HIV, and viral hepatitis infection in the same patient poses unique challenges to clinicians and public health officials. A major concern is that such patients may acquire clinical hepatitis during treatment with the first-line TB or HIV regimens available in most of the world. One study in HIV-infected TB patients demonstrated a doubling in liver enzyme levels in 14% of patients with HBV and 12% of patients with HCV infection, although few had symptomatic hepatitis.[10] Another small study found that 5 of 11 (45%) patients with both HCV and HIV infection developed drug-induced hepatitis during TB treatment; the risk of hepatitis was over 3 times greater than in those with HIV infection alone and over 14 times greater than in those with neither HIV nor HCV infection.[11]

Thailand is one of 22 World Health Organization (WHO)-designated "high burden" TB countries and has a generalized HIV epidemic.[12,13] Over 15% of TB patients in Thailand have HIV infection.[14] Among blood donors born before the introduction of universal hepatitis B vaccination in 1992, the percentage of Thais with hepatitis B surface antigen (HBsAg) and antibodies to HCV (anti-HCV) ranged from 4% to 13%[15] and 1% to 4% [15-17], respectively. Little is known about the burden of HBV and HCV infection in HIV-infected TB patients, or the impact of these infections on TB treatment outcomes in Thailand. We conducted an observational study in four provinces in Thailand to evaluate the prevalence of and risk factors for HBsAg and/or anti-HCV reactivity among HIV-infected TB patients undergoing TB treatment. We also evaluated the impact of HBV and HCV infections on adverse events during TB treatment and on final TB treatment outcome.

## Methods

### Study setting and population

We conducted a multi-center, observational, prospective cohort study among HIV-infected TB patients at public TB treatment facilities in Bangkok, Phuket, and Ubon Ratchathani provinces and at the national infectious diseases disease referral hospital (Bamrasnaradura Infectious Diseases Institute) in Nonthaburi province from May 2005 to September 2006. The study population included adults aged ≥ 18 years with documented HIV infection who were diagnosed with active TB disease according to national TB program guidelines[18], registered for TB treatment at one of the participating facilities, and received anti-TB therapy (for this episode of TB) for <4 weeks before study enrollment. We excluded prisoners and pregnant women. Patients with previous history of TB treatment were eligible for the study. Patients providing written informed consent were followed from TB treatment initiation to the end of TB treatment. For this study, patients received usual care for TB, HIV, and other diseases, and no health-related interventions were performed. In Thailand, patients with no prior history of TB treatment usually receive isoniazid, rifampin, ethambutol, and pyrazinamide. When prescribed anti-retroviral therapy, HIV-infected patients usually receive stavudine, lamivudine, and nevirapine; in patients with TB, efavirenz is recommended as a substitute for nevirapine.

### Data collection and laboratory studies

Patients had three study visits during TB treatment: at the beginning of treatment, at the end of the intensive phase of TB treatment (usually two months into treatment), and at the end of TB treatment (usually six months after treatment initiation). At the beginning of treatment, patients were interviewed using standardized study forms that asked about demographic characteristics, past and present medical history, knowledge and attitudes related to TB and HIV, and sex and drug use history. At every study visit, patients received a physical examination and were asked about medications taken and any adverse events experienced since their previous visit. Medical records were reviewed for results of examinations or tests performed between study visits. Adverse events were defined as any health-related problem that occurred during study follow-up, that was plausibly related to TB or HIV treatment, and that necessitated a physician evaluation. For adverse events, a definition of "liver disease" was applied to any patient who was diagnosed by a physician has having hepatitis, jaundice, or cirrhosis.

At the beginning of treatment, blood samples were tested for HBsAg and anti-HCV. No other tests of viral hepatitis infection were performed. These serologic tests for viral hepatitis markers were performed at the health facility enrolling the patient or, if necessary, at a centralized government laboratory in each respective province. For HBsAg, the following assays were used: Enzygnost (Dade Behring, Germany); AxSym HBsAg, version 2 (Abbott, Abbott Park, USA). For anti-HCV, the following assays were used: anti-HCV ELISA (Abbott, Abbott Park, USA); AxSym, version 3.0 (Abbott, Abbott Park, USA); HCV Tri-Dot (J. Mitra, India). In addition, blood samples were tested for aspartate aminotransferase (AST), alanine aminotransferase (ALT), total bilirubin (BR), complete blood count, and CD4+ T lymphocyte (CD4) cell count at the beginning of treatment. In these laboratories, the upper limit of normal for AST was 35 units/L, ALT 33 units/L, and total bilirubin 1 mg/dL. Sputum and specimens from extra-pulmonary sites were collected for acid fast bacilli (AFB) smear, mycobacterial culture, identification, and drug-susceptibility testing.

TB treatment outcomes were assessed at the end of TB treatment according to the national TB program and WHO guidelines; successful TB treatment comprised both cure and completed treatment.[18] For patients recorded as defaulting during TB treatment, we reviewed government vital registration data to determine whether patients died within 90 days of stopping TB treatment; such patients were re-classified as deaths during TB treatment.

### Statistical analysis

We calculated a TB knowledge score as the sum of correct answers to seven questions about TB and TB treatment; these questions were derived from information routinely provided to patients as part of the national TB program. High TB knowledge was defined as having a TB knowledge score equal to or greater than the median score of the study population. Similarly, we calculated a TB stigma score as the sum of four questions about TB stigma that a patient answered affirmatively (e.g., "Would you share a meal with someone with TB?"). Low TB stigma was defined as having a TB stigma score less than the median score of the study population.

We calculated proportions to describe demographic characteristics, clinical features, and adverse events. We analyzed groups stratified by viral hepatitis marker (HBsAg, anti-HCV, and both HBsAg and anti-HCV). However, patients were not broken down into 3 mutually exclusive categories: a patient who was positive for both markers was in the HBsAg analysis, the HCV analysis, and the "both" HBsAg and anti-HCV analysis. To identify characteristics predictive of these viral hepatitis markers, we first identified factors associated with each marker at p = 0.20 in univariate analysis and then constructed multivariate logistic regression models. For each multivariate analysis, we fitted a full model and a parsimonious model using a backward stepwise variable selection procedure. Findings from both models were similar; therefore, we reported estimates from the full model. There were 732, 736, and 740 patients included in the HBsAg, anti-HCV, and both HBsAg and anti-HCV analyses, respectively.

To determine whether different viral hepatitis markers were associated with different TB treatment outcomes, we analyzed the likelihood of death or a composite end point of death and default vs. TB treatment success, adjusted for factors associated with TB treatment outcome at p = 0.20 in univariate analysis. We fitted full and parsimonious models. Findings from both models were similar; we reported the estimates from the former. A subset of patients with culture-confirmed TB (defined as having any specimen culture-positive for MTB) was also analyzed.

For analysis of risk factors for viral hepatitis or the outcomes of TB treatment, we excluded 17 patients with unavailable viral hepatitis serology test results. In all analyses, we defined a two-sided p-value of ≤ 0.05 as statistical significance. We performed all analyses using Stata software version 8.0 (StataCorp LP, College Station, TX, U.S.A.).

### Ethical review

This study was approved by the ethical review committees of the Bangkok Metropolitan Administration, the Thailand Ministry of Public Health, and the U.S. Centers for Disease Control and Prevention.

## Results

### Characteristics and clinical features

**Figure 1:**
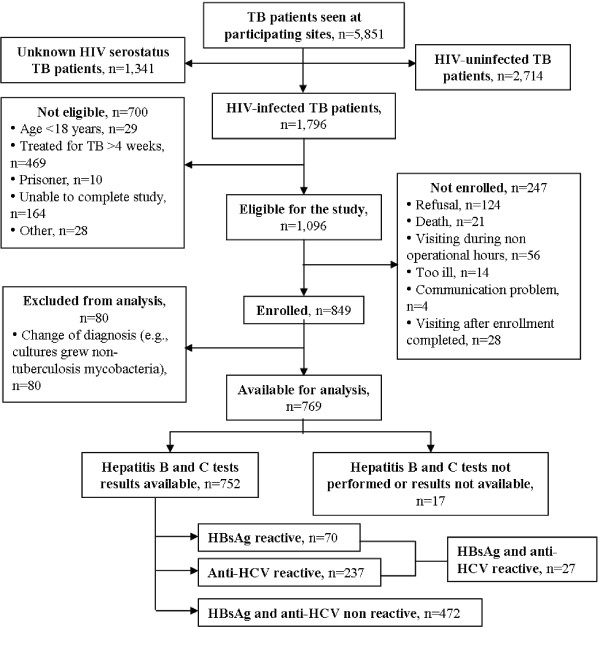
Enrollment of HIV-infected TB patients in the study.

We enrolled 849 HIV-infected TB patients in the study. Excluding those whose diagnosis of TB was subsequently changed (e.g., cultures grew non-tuberculosis mycobacteria), 769 (91%) were retained in the analysis (Figure 1). Of these, 752 (98%) had serologic tests for viral hepatitis infection. Seventy (9%) patients were reactive for HBsAg, 237 (31%) for anti-HCV, and 472 (63%) non-reactive for both HBsAg and anti-HCV. Twenty-seven (4%) patients were reactive for both HBsAg and anti-HCV. The median age of patients was 34 (interquartile range [IQR], 30–41) years, and most patients were male and employed (Table 1). Of the 769 patients, 461 (60%) had pulmonary TB, and 667 (87%) had not previously been treated for TB.

**Table 1:** Characteristics and clinical features of HIV-infected TB patients at the time of TB diagnosis, stratified by markers for viral hepatitis infection.

Characteristics and clinical features	All patients	Non-reactive for HBsAg and anti-HCV	Reactive for			Unknown
	(n = 769)	(n = 472)				(n = 17)
				
			Only HBsAg	Only anti-HCV	HBsAg and anti-HCV	
			(n = 43)	(n = 210)	(n = 27)	
**Characteristics**						
Age >34 years old	380 (49)	221 (47)	21 (49)	115 (55)	12 (44)	11 (65)
Male	538 (70)	294 (62)	32 (74)	175 (83)	25 (93)	12 (71)
>6th grade education	300 (39)	182 (39)	20 (47)	83 (40)	9 (33)	6 (35)
Employed	452 (59)	298 (63)	28 (65)	103 (49)	16 (59)	7 (41)
Single	237 (31)	125 (27)	14 (33)	83 (40)	10 (37)	5 (29)
Body mass index <18.5	443 (58)	270 (57)	24 (56)	116 (55)	21 (78)	12 (71)
Low TB knowledge	592 (77)	369 (78)	27 (63)	162 (77)	21 (78)	13 (77)
High TB stigma	500 (65)	294 (62)	26 (61)	146 (70)	20 (74)	14 (82)
Live in Bangkok	177 (23)	37 (8)	4 (9)	122 (58)	13 (48)	1 (6)
**TB disease classification**						
Pulmonary TB	461 (60)	261 (55)	21 (49)	146 (70)	21 (78)	12 (71)
Smear positive	283 (37)	169 (36)	13 (62)	80 (38)	11 (52)	10 (59)
Abnormal chest x-ray	415 (54)	233 (49)	17 (81)	134 (64)	20 (95)	11 (65)
Extra-pulmonary TB	230 (30)	168 (36)	16 (37)	37 (18)	5 (19)	4 (24)
Pulmonary and extra-pulmonary TB	78 (10)	43 (9)	6 (14)	27 (13)	1 (4)	1 (6)
**TB treatment**						
Registered for TB treatment as new case	667 (87)	424 (90)	35 (81)	173 (82)	19 (70)	16 (94)
Received standard or extended HRZE	665 (87)	430 (91)	35 (81)	168 (80)	17 (63)	15 (88)
DOT by healthcare worker or village health volunteer	246 (32)	100 (21)	17 (40)	107 (51)	14 (52)	8 (47)
**Drug and alcohol use, and incarceration history**						
Currently smoke	205 (27)	94 (20)	10 (23)	86 (41)	10 (37)	5 (29)
History of alcohol use	538 (70)	317 (67)	30 (70)	159 (76)	20 (74)	12 (71)
History of methamphetamine use	304 (40)	146 (31)	11 (26)	128 (61)	15 (56)	4 (24)
History of marijuana use	267 (35)	118 (25)	11 (26)	120 (57)	13 (48)	5 (29)
History of ketamine use	26 (3)	11 (2)	1 (2)	11 (5)	1 (4)	2 (12)
History of ecstasy use	32 (4)	16 (3)	1 (2)	12 (6)	1 (4)	2 (12)
History of sleeping pill use	138 (18)	56 (12)	9 (21)	64 (31)	7 (26)	2 (12)
History of inhalant use	130 (17)	53 (11)	8 (19)	57 (27)	9 (33)	3 (18)
History of injection drug use	199 (26)	43 (9)	4 (9)	134 (64)	15 (56)	3 (18)
History of incarceration	303 (39)	122 (26)	17 (40)	144 (69)	16 (59)	4 (24)
**High-risk sexual practices**						
Have >4 sex partners in past 6 months	9 (1)	5 (1)	1 (2)	2 (1)	1 (4)	0 (0)
Not always use condom*	247 (74)	151 (73)	13 (59)	67 (77)	6 (67)	10 (100)
Have sex with a sex worker in past 6 months*	29 (4)	17 (4)	3 (7)	7 (4)	2 (7)	0 (0)
Men who have sex with men	42 (8)	25 (9)	7 (22)	6 (4)	2 (8)	2 (17)
**Laboratory studies**						
Aspartate aminotransferase ≥ 120 units/L	67 (9)	35 (7)	7 (16)	18 (9)	4 (15)	3 (18)
Alanine aminotransferast ≥ 165 units/L	17 (2)	10 (2)	2 (5)	3 (1)	1 (4)	1 (6)
Bilirubin >2 mg/dL	77 (10)	50 (11)	6 (14)	14 (7)	4 (15)	3 (18)
CD4+ T-lymphocyte* (cells/μL), median (range)	63 (0–93)	53 (0–750)	41 (1–675)	76 (0–823)	101 (1–893)	139 (18–552)
**Medicines taken during TB treatment**						
Co-trimoxazole	665 (87)	411 (87)	39 (91)	181 (86)	22 (82)	12 (71)
Fluconazole	454 (59)	309 (66)	27 (63)	100 (48)	11 (41)	7 (41)
Anti-retroviral therapy	328 (43)	247 (52)	25 (58)	48 (23)	5 (19)	3 (18)

TB, tuberculosis; HIV, human immunodeficiency virus; HRZE, isoniazid, rifampin, pyrazinamide, ethambutol; DOT, directly observed therapy; HBsAg, hepatitis B surface antigen; HCV, hepatitis C virus

*Among those with available data/results

The median AST among patients reactive to HBsAg, anti-HCV, both HBsAg and anti-HCV, and neither were 58 (IQR, 35–92), 40 (IQR, 30–75), 59 (IQR, 36–105), and 58 (IQR, 40–101) units/L, respectively. The median ALT among patients reactive to HBsAg, anti-HCV, both HBsAg and anti-HCV, and neither were 33 (IQR, 22–54), 28 (IQR, 17–50), 37 (IQR, 20–63), and 37 (IQR, 24–52) units/L, respectively. The median bilirubin level among patients reactive to HBsAg, anti-HCV, both HBsAg and anti-HCV, and neither were 0.6 (IQR, 0.4–1.1), 0.5 (IQR, 0.3–0.9), 0.6 (IQR, 0.4–1.3), and 0.7 (IQR, 0.5–1.1) mg/dL, respectively. Of the 759 with available liver function test results, elevated levels were found in 18/70 (26%) patients with HBsAg (p = 0.02 compared to non-reactive), 37/236 (16%) patients with HCV (p = 0.93 compared to non-reactive), 6/27 (22%) patients with both (p = 0.42 compared to non-reactive), and 69/465 (15%) patients that were non-reactive.

### Risk factors for HBsAg and/or anti-HCV reactivity

Factors independently associated with HBsAg reactivity included being a man who had sex with men (adjusted odds ratio [AOR], 2.1; 95% confidence interval [CI], 1.1–4.3) and having low TB knowledge (AOR, 1.8; 95% CI, 1.0–3.0; Table 2.1). Factors independently associated with anti-HCV reactivity were being unemployed (AOR, 1.7; 95% CI, 1.1–2.5), having high TB stigma (AOR, 1.7; 95% CI, 1.0–2.7), currently smoking (AOR, 1.7; 95% CI, 1.0–2.9), having injection drug use history (AOR, 12.8; 95% CI, 7.0–23.2), and living in Bangkok (AOR, 15.8; 95% CI, 9.4–26.5; Table 2.2). Two factors were independently associated with having both HBsAg and anti-HCV reactivity: having injection drug use history (AOR, 3.2; 95% CI, 1.0–9.8) and living in Bangkok (AOR, 2.7; 95% CI, 1.1–6.6; Table 2.3).

**Table 2:** Univariate and multivariate logistic regression analyses of risk factors for viral hepatitis infections among HIV-infected TB patients.

1. Risk factors for HBsAg reactive (n = 732)								
Risk factors	OR	95% CI		p	AOR	95% CI		p
						
		L	U			L	U	

Male	2.0	1.1	3.7	0.03	1.6	0.8	3.1	0.16
Man having sex with men	2.3	1.2	4.6	0.02	**2.1**	**1.1**	**4.3**	**0.04**
Low TB knowledge	1.6	0.9	2.8	0.08	**1.8**	**1.0**	**3.0**	**0.04**
History of inhalant use	1.7	0.9	3.0	0.08	1.4	0.7	2.7	0.29
History of incarceration (jail)	1.4	0.9	2.3	0.20	1.2	0.7	2.0	0.62
Had >4 sex partners in past 6 months	2.8	0.6	13.9	0.20	1.9	0.4	9.4	0.46

2. Risk factors for anti-HCV reactive (n = 736)								

Risk factors	OR	95% CI		P	AOR	95% CI		p
						
		L	U			L	U	

Male	3.1	2.1	4.7	<0.01	1.4	0.8	2.5	0.25
Age >34 years	1.3	1.0	1.8	0.09	1.2	0.8	2.0	0.37
Unemployed	1.7	1.3	2.5	<0.01	**1.7**	**1.1**	**2.5**	**0.03**
High TB stigma	1.4	1.0	2.0	0.04	**1.7**	**1.0**	**2.7**	**0.04**
Currently smoke	2.7	1.9	3.8	<0.01	**1.7**	**1.0**	**2.9**	**0.04**
History of alcohol use	1.5	1.1	2.1	0.03	1.0	0.6	1.7	0.92
History of methamphetamine use	3.6	2.6	5.0	<0.01	0.7	0.4	1.3	0.32
History of marijuana use	3.9	2.8	5.5	<0.01	1.4	0.8	2.5	0.30
History of ketamine use	2.3	1.0	5.1	0.05	0.9	0.2	4.2	0.86
History of ecstasy use	1.7	0.8	3.6	0.15	1.1	0.3	4.9	0.88
History of sleeping pill use	3.0	2.1	4.4	<0.01	1.1	0.6	2.0	0.84
History of inhalant use	2.9	2.0	4.3	<0.01	0.9	0.5	1.7	0.68
History of injection drug use	17.2	11.5	25.6	<0.01	**12.8**	**7.0**	**23.2**	**<0.01**
History of incarceration (jail)	5.6	4.0	7.8	<0.01	1.3	0.8	2.3	0.35
Live in Bangkok	15.3	10.2	23.1	<0.01	**15.8**	**9.4**	**26.5**	**<0.01**

3. Risk factors for HBsAg and anti-HCV reactive (n = 740)								

Risk factors	OR	95% CI		P	AOR	95% CI		p
						
		L	U			L	U	

Male	5.6	1.3	23.8	0.02	3.2	0.7	14.4	0.14
History of methamphetamine use	2.1	0.9	4.6	0.07	0.7	0.2	2.2	0.54
History of marijuana use	1.9	0.9	4.2	0.11	0.8	0.3	2.4	0.69
History of inhalant use	2.7	1.2	6.2	0.02	1.9	0.7	5.4	0.21
History of injection drug use	4.5	2.0	10.2	<0.01	**3.2**	**1.0**	**9.8**	**0.05**
History of incarceration (jail)	2.3	1.0	4.9	0.04	1.0	0.3	3.3	0.97
Live in Bangkok	3.2	1.5	7.0	<0.01	**2.7**	**1.1**	**6.6**	**0.03**

TB, tuberculosis; HIV, human immunodeficiency virus; OR, odds ratio; AOR, adjusted odds ratio; CI, confidence interval; L, lower bound; U, upper bound; HBsAg, hepatitis B surface antigen; Variables for which p = 0.20 in univariate analyses were included in multivariate logistic regression analysis

### TB treatment and outcomes

A standard first-line TB regimen (two months of isoniazid, rifampin, pyrazinamide, and ethambutol followed by at least four months of isoniazid and rifampin) was used in 665 (87%) patients. Co-trimoxazole, fluconazole, and anti-retroviral therapy (ART) were taken by 665 (86%), 454 (59%), and 328 (43%), respectively, during TB treatment. The total follow-up time was 428 person-years. Four hundred and ninety-three patients (64%) were successfully treated (Table 3). Of the remaining patients, 6 (1%) failed treatment, 130 (17%) died, 65 (9%) defaulted, 70 (9%) transferred out, and 5 (1%) were still on treatment at the time follow-up ended.

**Table 3:** Most common adverse events during TB treatment and treatment outcomes among HIV-infected TB patients, stratified by markers for viral hepatitis infections.

Adverse events and treatment outcomes	All patients (n = 769)	Non-reactive for HBsAg and anti-HCV (n = 472)	Reactive for			Unknown (n = 17)
				
			Only HBsAg (n = 43)	Only anti-HCV (n = 210)	HBsAg and anti-HCV (n = 27)	
	
	n (%)	n (%)	n (%)	n (%)	n (%)	n (%)
**Adverse events**						
Rash	116 (15)	69 (15)	7 (16)	32 (15)	4 (15)	4 (24)
Immune reconstitution inflammatory syndrome*	16 (7)	12 (7)	0 (0)	2 (6)	1 (13)	1 (100)
Liver disease	41 (5)	23 (5)	3 (7)	12 (6)	2 (7)	1 (6)
Diarrhea	36 (5)	22 (5)	2 (5)	7 (3)	2 (7)	3 (18)
Pneumonia including PCP	30 (4)	15 (3)	2 (5)	11 (5)	1 (4)	1 (6)
Meningitis	20 (3)	11 (2)	2 (5)	6 (3)	1 (4)	0 (0)
HIV wasting syndrome	19 (3)	15 (3)	0 (0)	1 (1)	1 (4)	2 (12)
Cryptococcosis	16 (2)	8 (2)	0 (0)	8 (4)	0 (0)	0 (0)
Herpes zoster	12 (2)	5 (1)	2 (5)	4 (2)	1 (4)	0 (0)
Recurrent upper respiratory infections or sinusitis	7 (1)	5 (1)	0 (0)	0 (0)	2 (7)	0 (0)
**Treatment outcomes**						
Cure	210 (27)	133 (28)	7 (16)	63 (30)	6 (22)	1 (6)
Complete	283 (37)	190 (40)	16 (37)	65 (31)	9 (33)	3 (18)
Failure	6 (1)	3 (1)	1 (2)	2 (1)	0 (0)	0 (0)
Die	130 (17)	72 (15)	5 (12)	37 (18)	7 (26)	9 (53)
Default	65 (9)	34 (7)	8 (19)	20 (10)	1 (4)	2 (12)
Transfer out	70 (9)	37 (8)	5 (12)	22 (11)	4 (15)	2 (12)
On treatment	5 (1)	3 (1)	1 (2)	1 (1)	0 (0)	0 (0)

TB, tuberculosis; HIV, human immunodeficiency virus; HBsAg, hepatitis B surface antigen; HCV, hepatitis C virus; PCP, pneumocystis carinii pneumonia.

*Among 237 patients eligible to be evaluated only.

At least one adverse event occurred in 236 (31%) patients. The most common adverse events occurring during TB treatment (Table 3) were rash (15%), followed by immune reconstitution inflammatory syndrome (7%), liver disease (5%), diarrhea (5%), pneumonia (4%), meningitis (3%), HIV wasting syndrome (3%), cryptococcosis (2%), herpes zoster (2%), and recurrent upper respiratory infections or sinusitis (1%).

### Impact of viral hepatitis on adverse events and treatment outcomes

HIV wasting syndrome and upper respiratory infections or sinusitis occurring during TB treatment were the only health conditions significantly associated with markers of viral hepatitis infection (p = 0.02 and p = 0.05, respectively). Upper respiratory infections or sinusitis was reported by 7% of patients reactive to HBsAg and anti-HCV, 1% of patients non-reactive to any viral markers, and none in other groups. HIV wasting syndrome was reported in nearly 12% of patients with missing viral hepatitis markers, but in 4% of all other groups.

We analyzed the odds of death compared with successful TB treatment, stratified by markers of viral hepatitis infection and adjusted for factors known to be associated with TB treatment outcome. There was no difference in the odds of death for patients HBsAg reactive, anti-HCV reactive, or HBsAg and anti-HCV reactive, compared with patients non-reactive to any viral marker (Table 4.1). The findings were not significantly different when we analyzed all TB patients and only culture-confirmed patients. Factors adjusted for in this analysis included TB disease severity, HIV disease severity (i.e., CD4 count at time of enrollment), co-trimoxazole use, fluconazole use, anti-retroviral use, directly observed therapy use, hospitalization at enrollment, and previous TB treatment.

**Table 4:** Adjusted odds ratio for death and/or default outcomes among HIV-infected TB patients.

1. Those with cure, complete, death outcomes								
Comparison groups versus non-reactive	All patients				Culture-confirmed TB			
	n	AOR	95% CI		n	AOR	95% CI	
			L	U			L	U
HBsAg +	385	1.0	0.3	3.3	188	0.5	0.1	4.9
Anti-HCV +	504	1.1	0.6	1.9	253	1.4	0.6	3.6
HBsAg and anti-HCV +	374	2.1	0.6	7.6	185	4.8	0.5	42.9

2. Those with cure, complete, death, and default outcomes								
Comparison groups versus non-reactive	All patients				Culture-confirmed TB			
	n	AOR	95% CI		n	AOR	95% CI	
			L	U			L	U

HBsAg +	420	**2.7**	**1.1**	**6.4**	207	**5.5**	**1.4**	**21.9**
Anti-HCV +	549	1.2	0.8	2.0	281	**2.0**	**1.0**	**4.0**
HBsAg and anti-HCV +	401	1.4	0.4	4.7	198	1.7	0.3	11.2

+ sign indicates reactive; bold type indicates statistical significance (p < 0.05); TB, tuberculosis, HIV, human immunodeficiency virus; AOR, adjusted odds ratio; CI, confidence interval; L, lower bound; U, upper bound; HBsAg, hepatitis B surface antigen; HCV, hepatitis C virus, N, number of patients included in the model.

*Adjusted odds ratios from multivariate logistic regression analyses adjusted for TB disease severity, HIV disease severity, co-trimoxazole use, fluconazole use, anti-retroviral use, whether or not received directly observed therapy, hospitalized at enrollment and been treated for TB previously

We analyzed the odds of either death or default compared with successful TB treatment, stratified by viral hepatitis markers and adjusted for factors known to be associated with TB treatment outcome as noted above. Among patients HBsAg reactive, the odds of death or default was 2.7 (95% CI, 1.1–6.4) compared with patients non-reactive to any viral marker. This difference was also seen in the subset of culture-confirmed TB patients (Table 4.2). The odds of death or default was also increased in patients anti-HCV reactive, but this was only found in the subset of patients with culture-confirmed TB. Adjusting for injection drug use did not change the findings from these analyses.

## Discussion

Among HIV-infected TB patients living in Thailand, markers of viral hepatitis infection, particularly hepatitis C virus, were common and strongly associated with known behavioral risk factors. Viral hepatitis markers were not strongly associated with death or the development of clinical hepatitis during TB treatment.

The rate of HBsAg seropositivity in HIV-infected TB patients was similar to that found in the general Thai population.[15] In future years, the rate of HBV infection is likely to decline considerably, because universal hepatitis B childhood vaccination was integrated into the national expanded program on immunization in 1992.[16] Among HIV-infected TB patients, the strongest association with HBV infection was being a man having sex with men. In contrast to previous studies, we did not find an association between high-risk sexual practices (e.g., not using a condom, multiple partners) and HBsAg seropositivity, possibly because our sexual history questions probed only recent behavior and patients were generally quite ill.[19,20] Men who have sex with men are at high risk of HIV in Thailand, and the prevalence of HIV infection has risen dramatically in recent years.[21] This population, therefore, will increasingly require healthcare personnel sufficiently trained and equipped to manage concomitant HIV, TB, and viral hepatitis infection. We found that HBsAg seropositive patients were more likely to have elevated liver function enzymes, but, in contrast to studies in other settings, we found that TB treatment was well-tolerated, with rates of acquired hepatitis and other adverse events similar to patients not HBsAg seropositive.[22,23] Rates of default, however, were higher in this population, suggesting that further research is needed to explore whether medication intolerance and occult hepatitis could be factors leading to TB treatment default.

Antibodies to HCV were found in nearly one-third of patients, and nearly two-thirds of these cases reported a history of injection drug use. Other independent risk factors included living in Bangkok and smoking; given the known association between HCV infection and injection drug use[24], it is possible that living in Bangkok and smoking were surrogate markers for patients that did not accurately disclose their injection drug use history. We found that TB patients with HCV and HIV infection had adverse event rates and treatment outcomes no worse than TB patients with only HIV infection. Further research is needed to confirm this finding in other countries and in populations whose HCV infection is better characterized through measurement of HCV viral load, HCV genotype, and pathological evidence of liver disease.

Our study is subject to important limitations. First, we only performed limited laboratory testing on patients. Confirmatory tests, other serological markers of viral hepatitis, and viral load levels were not performed. Only a small proportion of those who become HBV infected will remain HBsAg positive; thus, factors associated with HBsAg positivity may be risk factors for HBV infection or determinants of chronic HBV disease. Serologic testing was also done in different laboratories using different commercial assays. Second, patients were only required to have liver function tests measured at the beginning of treatment. Our estimates of acquired hepatitis were based on persons who remained on TB treatment, had symptoms, sought care, and had a clinician diagnose clinical hepatitis. The incidence of clinical hepatitis developing during TB treatment, therefore, could be substantially higher than what we have reported, given the large number of patients that defaulted, transferred out, and died during treatment. The same limitation applies to our ascertainment of all adverse events. Finally, our sample size of patients may have been too small to detect low levels of hepatotoxicity, and the population we studied may not be representative of all HIV-infected TB patients in Thailand.

## Conclusion

The global burden of TB has risen dramatically in the past ten years due to the HIV epidemic. While HCV rates have similarly increased, the impact of viral hepatitis on the TB/HIV syndemic is less clear. This study provides important insight into the burden of viral hepatitis infection among HIV-infected TB patients living in Thailand, and demonstrates the need for further epidemiologic and clinical studies to optimize the management of this complicated medical condition.

## Competing interests

The authors declare that they have no competing interests.

## Authors' contributions

CS designed the study, collected data, and helped draft the manuscript. WK analyzed data and helped draft the manuscript. DP, PC, PL, LS, SN, SA, SL, WS, and CB helped design the study and collect data. JKV designed the study, analyzed data, and drafted the manuscript. All authors revised the draft manuscript and then approved the final version.

## Pre-publication history

The pre-publication history for this paper can be accessed here:


